# Blood group antigens and malaria susceptibility

**DOI:** 10.3389/fcimb.2025.1727335

**Published:** 2025-12-10

**Authors:** Fang-Fang Liu, Jing Liang, Ming Gao, Ke Li

**Affiliations:** 1 Department of Pathology, The Central Hospital of Wuhan, Tongji Medical College, Huazhong University of Science and Technology, Wuhan, China; 2 School of Life and Health Sciences, Hainan Province Key Laboratory of One Health, Collaborative Innovation Center of One Health, Hainan University, Haikou, Hainan, China; 3 Department of Blood Transfusion, Tongji Hospital, Tongji Medical College, Huazhong University of Science and Technology, Wuhan, China

**Keywords:** blood group systems, malaria, blood group antigens, blood group genes, malaria susceptibility

## Abstract

Blood-group antigens are increasingly recognized as key modulators of malaria susceptibility. This review synthesizes current evidence on how erythrocyte surface molecules shape parasite invasion, cytoadherence, immune evasion, and disease severity. We focus on a curated set of systems, ABO, Duffy, MNS, Gerbich, Knops, Diego, and others, and outline their molecular mechanisms affecting malaria. Population heterogeneity, parasite-host adaptations/counter-adaptations, and clinical implications for risk stratification, transfusion safety, and vaccine design are also discussed.

## 1 Introduction

Malaria remains one of the most devastating infectious diseases globally, with the World Malaria Report 2024 documenting 263 million cases and 597,000 deaths in 2023, predominantly affecting children under five in sub-Saharan Africa, which bears 94% of the global burden (Venkatesan, 2025). Despite progress in control and treatment, challenges such as drug resistance, invasive vectors, and climate-driven transmission shifts persist. Host genetic factors are increasingly recognized as key determinants of disease outcomes, accounting for an estimated 25% of malaria progression risk (Mackinnon et al., 2005). Malaria susceptibility is influenced not only by genetic disorders affecting erythrocyte biology, such as sickle cell disease, G6PD deficiency, and alpha-thalassemia, but also by natural variations in key RBC physiological properties, such as hydration, membrane deformability, and volume (Malaria Genomic Epidemiology N, 2019; Ebel et al., 2021). Among these, erythrocyte blood group antigens—polymorphic surface molecules encoded by 48 internationally recognized systems (ISBT Working Party: Table of blood group systems v12.0 (31 May 2025)) —directly influence parasite invasion efficiency and the intracellular environment, thereby linking human genetic diversity to malaria outcomes (Cooling, 2015). This review synthesizes current evidence on blood group antigens as determinants of malaria susceptibility, focusing on their roles in Plasmodium invasion, immune evasion, and population genetics. By integrating molecular insights with epidemiological data, we aim to bridge foundational science with translational applications in vaccines, diagnostics, and transfusion medicine.

## 2 Overview of human red blood cell blood group systems

The ISBT recognizes 48 genetically distinct blood group systems as of May 2025, encompassing over 300 antigens primarily expressed on erythrocyte membranes. These systems are defined by polymorphisms in genes encoding transmembrane proteins, glycoproteins, and glycolipids that govern antigen specificity, with each system assigned a unique identifier (e.g., ABO: 001; Duffy: 008) and categorized based on molecular characteristics, chromosomal locations, and antigen polymorphisms (Reid et al., 2012).

**Figure 1:**
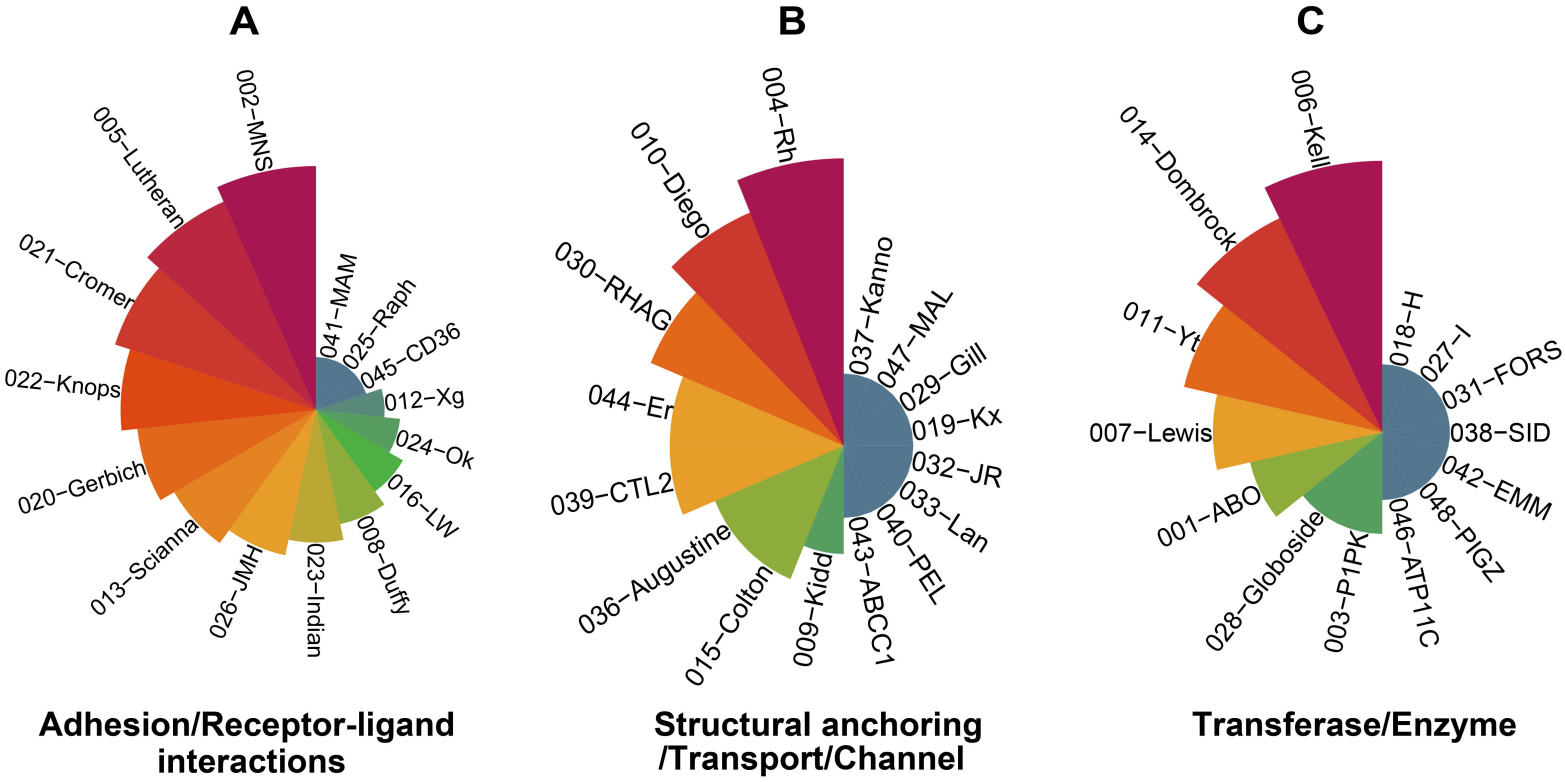
The functional classification of major human blood-group systems. **(A)** adhesion/receptor-ligand interactions. **(B)** structural anchoring/transport/channel. **(C)** transferase/enzyme. Each sector represents one blood-group system labeled “ISBT-system name”; The sector area is related to the number of antigens contained in the blood type system. The more antigens there are, the larger the sector area occupied by the blood type system.

Blood group antigens perform multiple essential biological functions, including structural anchoring, signal transduction, receptor-ligand interactions, adhesion, enzymatic effect, complement regulation, etc (Reid et al., 2012). Structural anchoring maintains membrane integrity through cytoskeletal linkages. Band 3 (Diego blood group system) tethers the lipid bilayer to spectrin-ankyrin complexes via its cytoplasmic domain, while glycophorin C (Gerbich blood group system) binds protein 4.1 to stabilize junctional complexes (Lux, 2016). Mutations in SLC4A1 (Diego blood group system) disrupt cytoskeletal cohesion, leading to hereditary spherocytosis (Paquette et al., 2015). Kell (Kell blood group system) glycoprotein functioning as a zinc-dependent endopeptidase that cleaves endothelin-3, thereby regulating vasoconstriction and blood pressure (Denomme, 2015). PIEZO1 (Er blood group system) modulates erythrocyte volume and deformability via its key role in regulating biomechanical properties and ion homeostasis under circulatory pressure (Ma et al., 2018). Rh proteins (Rh blood group system), together with RhAG (RHAG blood group system) and the scaffolding protein ankyrin-1, assemble into a multiprotein complex that shapes membrane curvature and preserves deformability (Ramsey, 2025); in Rh-null erythrocytes, loss of this complex yields pleomorphic stomatocytic-spherocytic cells, reflecting disrupted lipid asymmetry and destabilized membrane skeleton (Ramsey, 2025). Signal transduction roles include Duffy (DARC), which binds inflammatory chemokines (e.g., IL-8, CCL2, CCL5) to modulate leukocyte recruitment (Morein et al., 2020; Maheshwari et al., 2025), and CR1 (Knops blood group system), which accelerates C3b cleavage via factor I cofactor activity, limiting complement-mediated lysis (Smith et al., 2002). Enzymatic effect (glycosyltransferases and other catalytic enzymes) refers to the catalytic activities carried out by specific blood-group proteins. The most well-known ones are the ABO, Lewis, H and Kell blood group systems (Lee et al., 2000; Morgan and Watkins, 2000). Receptor–ligand interactions/adhesion underpin pathogen recognition: DARC serves as a receptor for Plasmodium vivax Duffy-binding protein (PvDBP) (Batchelor et al., 2011), while glycophorin A (MNS blood group system) facilitates P. falciparum invasion via EBA-175 binding (Tolia et al., 2005). ABO antigens influence pathogen or cell adhesion through glycan-mediated interactions with selectins and galectins (Stowell et al., 2010). CD44 (Indian blood group system) binds hyaluronic acid to mediate leukocyte homing and tumor metastasis, while its ectodomain shedding regulates inflammatory responses (Ponta et al., 2003). CD55 (Cromer blood group system) inhibits C3 convertase assembly, preventing accidental complement activation on host cells (Harris et al., 2007). **Figure 1** shows the main biological functional classification of the blood type systems.

Here, we mainly focus on seven blood type systems significantly associated with malaria, which are: ABO (ISBT 001), located on chromosome 9q34.2, encodes glycosyltransferases that synthesize carbohydrate antigens (A, B, H) on glycoproteins and glycolipids. The A and B antigens differ by a terminal sugar residue (N-acetylgalactosamine vs. galactose), while H antigen serves as the precursor structure (Stowell and Stowell, 2019). Duffy (ISBT 008), governed by the ACKR1 gene at 1q23.2, encodes a chemokine receptor protein (DARC) with seven transmembrane domains. Its antigens (Fy^a^, Fy^b^) result from a single amino acid substitution (p.Gly42Asp) (Maheshwari et al., 2025). The MNS blood-group system (ISBT 002) maps to chromosome 4q31.21 and comprises the tightly linked genes GYPA, GYPB and GYPE, which encode the single-pass sialoglycoproteins glycophorin A (GPA), glycophorin B (GPB) and glycophorin E (GPE). GYPA and GYPB produce MN and Ss antigens, respectively, while GYPE encodes low-abundance variants (Castilho, 2019). Gerbich (ISBT 020) maps to 2q14.3 and involves glycophorin C/D (GYPC), which carries Ge antigens critical for membrane stability (Walker and Reid, 2010). Knops (ISBT 022) localizes to 1q32.2 and expresses antigens (e.g., Kn^a^, McC^a^) on complement receptor 1 (CR1), a glycoprotein regulating immune complex clearance (Moulds, 2019). Diego (ISBT 010) at 17q21.31 encodes band 3 protein (SLC4A1), an anion exchanger bearing Diego (Di^a^) and Wright (Wr^a^) antigens (Figueroa, 2013). The Er blood group system (ISBT 044) is encoded by the PIEZO1 gene located on chromosome 16q24.3. This gene encodes the PIEZO1 protein, a mechanosensitive ion channel that serves as the carrier for Er antigens on erythrocytes. Five high-prevalence Er antigens (Era, Erb, Er3, Er4, Er5) have been characterized, with specific missense mutations in PIEZO1 (including R2456H and L2618M) resulting in altered Er antigen expression profiles (Karamatic Crew et al., 2023).

## 3 Epidemiological profile of blood group polymorphisms and malaria susceptibility

Blood group polymorphisms exhibit significant epidemiological associations with malaria susceptibility across diverse endemic regions. The Duffy-negative phenotype (Fy(a-b-)), prevalent in >95% of West/Central African populations due to homozygosity for the FYB^ES^ allele, confers strong protection against P. vivax invasion (Langhi and Bordin, 2006). This explains the historical absence of P. vivax in these regions, though Duffy-independent strains now challenge this protection in Madagascar and Ethiopia (Lo et al., 2021; Bouyssou et al., 2023). For P. falciparum, ABO blood groups substantially influence severe disease risk: blood group O reduces severe malaria odds by 66% (OR 0.34, 95% CI 0.19–0.61) compared to non-O groups (Rowe et al., 2007); Non-O homozygous genotypes (AA/BB) show higher severe malaria susceptibility than heterozygotes (AO/BO) (Opi et al., 2023).

Geographical heterogeneity modulates these associations. In Ghana and Nigeria, blood group O individuals paradoxically demonstrate higher P. falciparum infection rates (Opara et al., 2006; Yeda et al., 2022), suggesting protection targets disease severity rather than initial infection. The role of CR1 density polymorphisms in malaria susceptibility is complex and appears to be highly dependent on the epidemiological context. Specifically, the low-expression CR1 alleles yield conflicting data: they are associated with an increased risk of severe malaria in low-transmission settings but appear to be protective in high-transmission areas (Sinha et al., 2009; Cooling, 2015). This population-specific effect is clearly illustrated by polymorphisms of the Knops blood group system. For instance, the Sl2 low-expression allele reduces the risk of severe malaria in Kenya (a setting with historically high-to-moderate transmission) but shows no association in Gambia (where transmission intensity is generally lower) (Bellamy et al., 1998; Thathy et al., 2005). This contrast between Kenya and Gambia provides a compelling example of how the same allele can have divergent effects under different transmission pressures. Similarly, Gerbich-negative phenotypes (glycophorin C deficiency) reduce P. falciparum or P. vivax infection by >50% in Papua New Guinea, though clinical correlates require further quantification (Serjeantson, 1989; Patel SS. et al., 2004). A gain-of-function mutation in PIEZO1, E756del, is highly prevalent in African populations (with allele frequencies up to 30%) but is extremely rare elsewhere (Ma et al., 2018). This distinct geographic distribution is consistent with a selective advantage against Plasmodium falciparum. Indeed, case-control studies conducted in Gabon and Senegal have demonstrated that heterozygous carriers of the E756del mutation enjoy a significantly reduced risk of developing severe malaria, particularly cerebral malaria (Nguetse et al., 2020). These findings provide strong genetic and epidemiological evidence that the E756del variant confers a protective effect against the most lethal forms of the disease (Ebel et al., 2021).

No consistent epidemiological links exist for Rh blood groups. Meta-analyses of 36 studies found no significant risk difference between Rh-positive and Rh-negative individuals (Rattanapan et al., 2023). Likewise, a recent prospective cohort of 749 Moroccan soldiers under stringent mefloquine prophylaxis found no association between Rh status and malaria incidence, recurrence, or progression to severe disease (El Faria et al., 2025). Globally, host genetics accounts for ~25% of malaria progression risk, highlighting the cumulative impact of blood group variants (Mackinnon et al., 2005).

## 4 Molecular mechanisms of blood group genes/antigens in malaria pathogenesis

### 4.1 ABO blood group system

The ABO blood group system critically influences Plasmodium falciparum pathogenesis through terminal glycan structures on erythrocyte surface glycoproteins. The A and B antigens (GalNAcα1-3Gal and Galα1-3Gal, respectively) enhance P. falciparum erythrocyte membrane protein 1 (PfEMP1)-mediated rosetting by strengthening bonds between infected and uninfected erythrocytes via the N-terminal Duffy-binding-like (DBL) domain of PfEMP1 (Moll et al., 2015; Opi et al., 2023). Structural analyses confirm that PfEMP1-DBLα specifically recognizes the A-trisaccharide motif (GalNAcα1-3[Fucα1-2]Gal), thereby stabilizing rosettes and enhancing their capacity to cause microvascular obstruction under physiological shear stress (Vigan-Womas et al., 2012; McQuaid and Rowe, 2020). Importantly, PfEMP1 is not the sole mediator; other variant surface antigen (VSA) families contribute to rosetting. RIFIN proteins, for instance, can bind directly to the A-blood group antigen itself, providing an alternative, PfEMP1-independent mechanism for rosette formation (Goel et al., 2015). STEVOR proteins have also been functionally implicated in the rosetting phenotype, highlighting the multifaceted nature of this cytoadhesive phenomenon (Niang et al., 2014). In contrast, O-group erythrocytes lack A/B antigens, reducing rosette formation by 60–70% and conferring significant protection against severe malaria (OR = 0.34, 95% CI: 0.19-0.61) (Rowe et al., 2007). Non-O homozygous genotypes (AA/BB/AB) exhibit a much higher risk of severe malaria than heterozygous genotypes (AO/BO), demonstrating a clear gene-dosage effect (Opi et al., 2023). This risk stratification is further supported by population studies in the Democratic Republic of Congo, where A-blood group individuals showed a 3.3-fold higher incidence of severe malaria compared to O-group individuals (Tonen-Wolyec and Batina-Agasa, 2021).

### 4.2 Duffy blood group system

The Duffy antigen receptor for chemokines (DARC), encoded by the ACKR1 gene, serves as the primary receptor for Plasmodium vivax Duffy-binding protein (PvDBP). The invasion of Plasmodium vivax into human erythrocytes is mediated by the interaction between the parasite’s Duffy-binding protein (PvDBP) domain II and the host’s Duffy antigen receptor for chemokines (DARC). Specifically, PvDBP binds to the N-terminal extracellular region (residues 19-30) of DARC (Batchelor et al., 2014; Moskovitz et al., 2023). This interaction exhibits allelic specificity, with PvDBP having a higher affinity for the Fyb allele than the Fya allele, a difference attributed to amino acid polymorphisms at positions 42 and 89 of the DARC protein (Batchelor et al., 2014; Moskovitz et al., 2023). Critically, a promoter variant (−67T>C, rs2814778) results in the Fy(a−b−) phenotype, which ablates DARC expression on erythrocytes. This prevents P. vivax invasion and underlies the near absence of vivax malaria in West and Central Africa, where the Fy(a−b−) phenotype is present in over 95% of the population (Maheshwari et al., 2025). However, emerging evidence from Madagascar, Ethiopia, and Brazil indicates that P. vivax strains utilize alternative receptors (e.g., CD98hc (Malleret et al., 2021) or transferrin receptor 1 (Gruszczyk et al., 2018)) for Duffy-independent invasion, resulting in 8.8–15% infection rates among Fy(a−b−) individuals (Menard et al., 2010). Heterozygous Fy(a+b−) individuals show 30–80% reduced susceptibility due to decreased DARC density on erythrocytes (Kasehagen et al., 2007; King et al., 2011). Notably, P. falciparum invasion remains unaffected by Duffy status, as it relies on glycophorin-mediated pathways.

### 4.3 MNS and Gerbich blood group systems

Glycophorins serve as critical invasion receptors for P. falciparum merozoites. Its ligand, erythrocyte-binding antigen 175 (EBA-175), binds to the sialylated, O-linked glycan-rich N-terminal region of GYPA, with terminal sialic acid residues essential for high-affinity engagement and parasite invasion (Pisano et al., 1993; Tolia et al., 2005). Glycophorin B (GYPB) acts as the erythrocyte receptor for the P. falciparum ligand EBL-1; erythrocytes lacking GYPB (S-s-U- phenotype) are refractory to EBL-1-mediated invasion, although the parasite can compensate via alternative pathways (Mayer et al., 2009). Glycophorin C (GPC) is the principal erythrocyte receptor for the Plasmodium falciparum ligand EBA-140 (Maier et al., 2003). The Gerbich-negative (Ge (–)) phenotype, caused by a GYPC exon 3 deletion, abrogates this binding site. Consequently, the 3D7 parasite strain shows a ~40–60% reduction in invasion efficiency toward Ge (–) erythrocytes *in vitro*. Consistent with strong malaria-driven selection, the Ge (–) allele reaches a frequency of 46.5% in coastal Papua New Guinean populations where P. falciparum is hyperendemic (Maier et al., 2003; Jaskiewicz et al., 2018). The Dantu blood variant arises from a complex structural rearrangement (DUP4) that replaces full-length GYPB with two GYPA-GYPB hybrid genes. These hybrids encode a chimeric glycophorin whose extracellular GYPB domain is linked to GYPA transmembrane/cytoplasmic segments, raising erythrocyte membrane tension and stiffening the bilayer (Unger et al., 1987; Leffler et al., 2017). *In vivo* CHMI studies in Kenya demonstrate strong protection: none (0/30) of the Dantu carriers reached the predefined treatment threshold of 500 parasites/µl within 21 days, compared with 22.5% (25/111) of non-Dantu controls (Kariuki et al., 2023); these findings align with population-based data indicating a ~40% reduction in severe malaria risk associated with the Dantu variant (Leffler et al., 2017). Emerging evidence indicates that the Dantu blood-group variant confers malaria protection via a dual biomechanical mechanism: a structurally altered, hybrid glycophorin elevates erythrocyte membrane tension, thereby impeding merozoite invasion (Kariuki et al., 2020), while concomitant reductions in cell size and surface-adhesion receptors (e.g., GYPA, CR1) density attenuate PfEMP1-mediated rosette formation and subsequent microvascular sequestration (Kariuki et al., 2020; Carlier et al., 2024).

### 4.4 Diego blood group systems

The SLC4A1 gene encodes the erythroid anion exchanger Band 3, a protein critically involved in erythrocyte physiology and malaria pathogenesis. Beyond its role in anion transport and membrane skeletal organization, Band 3 serves as a key receptor for merozoite invasion. For Plasmodium falciparum, the merozoite surface protein EBA-175 specifically interacts with the extracellular domain of Band 3, a binding event crucial for successful parasite entry (Goel et al., 2003). A parallel mechanism exists in P. vivax, where the parasite ligand PvTRAg38 binds to Band 3, with a specific 12-amino-acid domain interacting with multiple extracellular loops of the receptor (Alam et al., 2016).

The malaria-protective trait known as Southeast Asian ovalocytosis (SAO) is underpinned by a 27-bp in-frame deletion in SLC4A1 (codons 400–408). This deletion, located in exon 11, truncates a highly conserved cytoplasmic–membrane boundary segment of Band 3. The resultant protein exhibits markedly reduced lateral mobility and increases erythrocyte rigidity through tighter ankyrin binding (Mirchev et al., 2011; Reithmeier et al., 2016). It is proposed that this structural alteration may also sterically hinder the binding of parasite ligands like EBA-175 and PvTRAg38 to Band 3, providing a dual protective mechanism by concurrently stiffening the erythrocyte and directly impeding invasion.

Critically, SAO is strongly associated with a reduced risk of cerebral malaria caused by Plasmodium falciparum. Population-based studies in Papua New Guinea and Indonesia demonstrate that heterozygous carriers experience significantly lower rates of severe malaria, although no single quantitative protection figure has been uniformly reported (Genton et al., 1995; Wilder et al., 2009). Notably, the deletion is embedded within a conserved haplotype that includes the “Memphis” missense variant (Lys56Glu), suggesting that additional functional alterations in band 3-mediated anion transport or membrane organization may further modulate the protective phenotype (Wilder et al., 2009).

### 4.5 Knops system (CR1)

The Knops blood group system is a blood group system based on complement receptor 1 (CR1/CD35) that was officially recognized by the ISBT in 1992 (Moulds, 2010). CR1 contains four tandem long homologous repeats (LHR-A, LHR-B, LHR-C, LHR-D), each harboring seven short consensus repeats. LHR-B and LHR-C constitute the principal binding sites for C3b and, to a lesser extent, C4b, whereas LHR-A preferentially binds C4b and LHR-D interacts with C1q, mannose-binding lectin and L-ficolin (Wymann et al., 2024). Engagement of C3b or C4b by LHR-B/C positions the convertase complexes so that CR1 accelerates their dissociation (decay-accelerating activity) and, together with factor I, catalyzes the proteolytic inactivation of C3b/C4b (cofactor activity) (Hardy et al., 2023). These concerted actions restrain excessive complement activation and enhance the clearance of immune complexes across the classical, lectin and alternative pathways. In Plasmodium falciparum malaria, CR1 serves as an adhesion receptor for P. falciparum erythrocyte membrane protein 1 (PfEMP1), promoting the formation of rosettes (clusters of infected and uninfected erythrocytes) that contribute to microvascular obstruction and severe disease (Deb et al., 2024). Rosette formation is associated with severe malaria. Low-CR1-expressing antigens impair rosette formation and thereby maybe reduce the risk of severe malaria. Variants such as Sl2 (p.R1601G) and McC^b^ (p.K1590E) within the Knops system substitute neutral glycine for basic arginine and acidic glutamic acid for basic lysine, respectively; these changes may stabilize CR1 on the erythrocyte surface via removing proteolytic cleavage sites and have been associated with higher CR1 levels in population studies (Moulds et al., 2001; Opi et al., 2016). Paradoxically, Kenyan children carrying the Sl2 and McC^b^ alleles display higher rather than lower CR1 densities yet show a lower incidence of cerebral malaria, suggesting that elevated CR1 levels may enhance immune-complex clearance or complement regulation without exacerbating rosetting (Thathy et al., 2005; Opi et al., 2016). However, population surveys in other regions have not replicated this protective trend, suggesting that the association may be influenced by both host genetic background, parasite ligand diversity, and additional environmental factors such as transmission intensity and co-infection patterns (Hansson et al., 2013; Tettey et al., 2015; Opi et al., 2018).

### 4.6 Er blood group system

The protective mechanism of the Er blood group system against malaria operates through precisely regulated biomechanical and biochemical pathways mediated by PIEZO1 gain-of-function mutations (Karamatic Crew et al., 2023; Lohia et al., 2023; Zhang et al., 2024). These mutations trigger a signaling cascade that stiffens the erythrocyte membrane by stabilizing its cytoskeleton. Consequently, the rigidified cell surface physically impedes merozoite invasion (Andolfo et al., 2023). Concurrently, the mutations disrupt intracellular ion homeostasis through sustained calcium influx, which activates the Gardos channel and induces potassium efflux, leading to cellular dehydration and reduced parasite growth efficiency (Nguetse et al., 2020; Karamatic Crew et al., 2023). Furthermore, elevated intracellular calcium levels impair parasite digestion of hemoglobin by inhibiting the parasite’s aspartic proteases (plasmepsins) and cysteine proteases (falcipains) (Ma et al., 2018; Lohia et al., 2023). This multi-layered defense strategy—combining enhanced mechanical resistance with disruption of the intracellular environment—represents an elegant evolutionary adaptation to malaria pressure, explaining the high prevalence of these PIEZO1 variants in endemic regions (Nguetse et al., 2020).

### 4.7 CD36, Ok, Cromer, Landsteiner-Wiener, Kx, ATP11C, and Colton blood group systems

In addition to the extensively characterized ABO, Duffy, MNS, Gerbich, Knops and Diego blood group systems, CD36, Ok, Cromer, Landsteiner-Wiener, Kx and ATP11C blood group systems also play significant roles in malaria infection and pathogenesis. They were first defined by serology alone, but each encodes a membrane protein (CD36, BASIGIN, CD55, ICAM4, XK and ATP11C) that is now known to double as a ligand, receptor or transporter exploited by malaria parasites. Emerging population-genetic, cellular, and structural studies reveal that common or rare alleles within these systems modulate parasite invasion, cytoadherence, cytokine balance and even red-cell deformability. In this section we synthesize these scattered findings and concisely summarize how these genes shape malaria susceptibility and severity (**Table 1**).

**Table 1:** Blood group systems with clear association with malaria.

ISBT no.	System name	Gene (Chromosome location)	CD numbers	Key antigens /phenotypes/variants	Malaria-relevant mechanisms
1	ABO	ABO (9q34.2)	/	A, B, H	Microvascular sequestration; rosetting
2	MNS	GYPA/GYPB/GYPE (4q31.21)	CD235a, CD235b	M/N, S/s; Dantu, S-s-U-	P. falciparum invasion receptor
8	Duffy	ACKR1 (1q23.2)	CD234	Fy(a), Fy(b); Fy(a–b–)	P. vivax invasion receptor
10	Diego	SLC4A1 (17q21.31)	CD233	Di(a), Di(b); SAO	RBC membrane rigidity
16	Landsteiner-Wiener	ICAM4 (19p13.2)	CD242	LW(a), LW(b)	Microvascular sequestration; rosetting
20	Gerbich	GYPC (2q14.3)	CD236	Ge2, Ge3, Ge4; Ge (–)	P. falciparum invasion receptor
21	Cromer	CD55 (1q32.2)	CD55	Cra, Tca, Dr(a)	Parasite internalization
22	Knops	CR1 (1q32.2)	CD35	Kn(a), Kn(b); Sl2, McCb	Rosetting
24	Ok	BSG (19p13.3)	CD147	Ok(a)	P. falciparum invasion receptor
44	Er	PIEZO1(16q24.3)	/	Era, Erb, Er3, Er4, Er5	Erythrocyte biomechanical properties and ion homeostasis
45	CD36	CD36 (7q21.11)	CD36	CD36 deficiency	Microvascular sequestration; phagocytosis

Each entry lists the ISBT number, gene locus, key antigens/variants, and the specific malaria-relevant mechanisms.

The CD36 receptor serves as a crucial adhesion molecule for Plasmodium falciparum-infected erythrocytes (IEs), facilitating sequestration in microvasculature through binding to the CIDRα domain of PfEMP1 (Mo et al., 2008). This interaction triggers endothelial activation and inflammatory responses, contributing to severe malaria pathology (e.g., plasma leakage and brain edema) (Trivedi and Chakravarty, 2022). Notably, CD36 deficiency or reduced surface expression confers partial protection against malaria by significantly decreasing IE cytoadherence (Bachmann et al., 2022). However, CD36 also mediates phagocytic clearance of IEs by macrophages and dendritic cells, highlighting its dual role in malaria immunity (Patel SN. et al., 2004; Gowda et al., 2013). Basigin (BSG, CD147) functions as an essential erythrocyte receptor for the P. falciparum Rh5 invasion protein (PfRh5) (Crosnier et al., 2011). The PfRh5-basigin interaction initiates the formation of a tight junction complex critical for merozoite entry into red blood cells (Muramatsu, 2012). Antibodies blocking this binding effectively neutralize parasite invasion across diverse strains (Crosnier et al., 2011; Wright et al., 2014). CD55 (decay-accelerating factor) facilitates parasite internalization during erythrocyte invasion (Egan et al., 2015). It stabilizes the moving junction structure between merozoite and host membrane after rhoptry discharge (Shakya et al., 2021). Notably, CD55-deficient erythrocytes exhibit significantly reduced invasion efficiency for multiple P. falciparum strains (Shakya et al., 2021), indicating its conserved role in the invasion of P. falciparum. ICAM4 interacts with endothelial αVβ3 integrins under static or low-shear conditions (Zennadi et al., 2004). This adhesion may promote IE sequestration and microvascular obstruction. Soluble ICAM4 peptides competitively inhibit IE-endothelial binding, suggesting therapeutic potential for reducing cytoadherence-related pathology (Bhalla et al., 2015). XK and ATP11C preserve membrane integrity: XK loss causes McLeod syndrome with acanthocytosis and reduced deformability that may impair merozoite entry (Roulis et al., 2018), while ATP11C-mediated phosphatidylserine flipping prevents premature PS exposure and macrophage clearance (Arashiki et al., 2016; Nagata et al., 2016; Shin and Takatsu, 2019). No population data link XK variants to malaria, yet CRISPR KEL-null erythrocytes show 60% invasion reduction (Kumari et al., 2025). and since XK is required for Kell surface stability, any XK disruption would compromise the Kell–XK complex and potentially replicate the protective phenotype. Thus, XK is an indirect but functionally relevant modulator of malaria susceptibility.

Except for the twelve blood-group systems described above, antigens or genes from additional systems, such as AQP1 (Colton) (Olivieri et al., 2021), ART4 (Dombrock) (Olivieri et al., 2021), BCAM (Lutheran) (Dalimot et al., 2022) and SMIM1 (Vel) (Aniweh et al., 2019), have also been implicated in malaria, yet systematic functional data remain unclear. Recently, our transcriptomic study of orthochromatic erythroblasts found that among 51 blood-group genes spanning all 45 ISBT-defined systems, 22 correlated significantly with P. falciparum infection status, and 11 (including AQP1, ART4, and BCAM) exhibited marked expression changes upon parasite infection (Liu and Li, 2024). These data suggest a broad, still under-appreciated, interplay between erythrocyte antigens and malaria that merits deeper investigation.

## 5 Conclusions and future perspectives

Over the past century, blood-group science has evolved from a practical tool for cross-matching transfusions into a molecular telescope for deciphering host–parasite co-evolution. Evolutionary pressure has been selected for erythrocyte variants that confer protection against malaria through distinct mechanisms. Mutations affecting surface proteins (e.g., Duffy, ABO) and cytoskeletal proteins (e.g., SLC4A1/Diego, PIEZO1/Er) impair parasite invasion, while hemoglobinopathies (e.g., sickle cell trait, thalassemia) and enzyme deficiencies (e.g., G6PD) disrupt intraerythrocytic parasite growth and enhance clearance (Ebel et al., 2021). These variations collectively reduce parasite biomass and severe malaria risk. For example, PfEMP1 expression is diminished in HbAS erythrocytes, reducing cytoadhesion (Cholera et al., 2008), while thalassemia is associated with decreased Duffy and CR1 expression, limiting invasion (Prajapati et al., 2019; Huang et al., 2025). However, validating putative additive effects like sickle cell trait and blood group O necessitates large-scale genetic studies, a challenging endeavor due to sample size requirements and population heterogeneity.

This review has systematically examined how ABO, Duffy, MNS, Gerbich, Knops, Diego, Er and other blood-group systems shape malaria susceptibility through antigenic variation, revealing two unifying themes. First, erythrocyte surface antigens orchestrate parasite invasion, cytoadherence, immune evasion and microvascular obstruction via dual “receptor-ligand adhesion” and “biomechanical-signaling” interfaces. Second, the geographic distribution of these antigens/variants mirrors malaria ecological niches, providing a quantifiable natural experiment in global populations.

Growing evidence underscores the critical role of erythropoiesis and the bone marrow microenvironment in malaria parasite biology, shifting focus from the established paradigm of mature erythrocytes in peripheral circulation (Venugopal et al., 2020). The expression profiles of many blood group antigens are not static but undergo dynamic changes during erythroid differentiation (Bony et al., 1999). A key example is the Duffy blood group system: individuals with the Duffy-negative phenotype (Fy(a–b–)) exhibit strong resistance to Plasmodium vivax infection of their mature erythrocytes, which lack the Duffy antigen/receptor for chemokines (DARC) (Langhi and Bordin, 2006). However, early erythroid progenitors and erythroblasts in these individuals frequently express DARC during maturation, enabling the bone marrow to serve as a hidden site for P. vivax infection, bypassing the resistance mechanism observed in peripheral blood (Bouyssou et al., 2023). In addition, both Plasmodium falciparum and P. vivax may also exploit stage-specific antigens—such as those of the Gerbich and Knops systems—to invade erythroblasts in the bone marrow. Future studies should map the developmental expression of blood group antigens and determine how Plasmodium species exploit these dynamic interfaces to define the genetic basis of susceptibility and resistance.

Blood group systems may constitute key genetic determinants for stratifying malaria risk and guiding targeted interventions. The ABO system significantly influences disease severity, wherein group O confers protection against severe malaria by impairing PfEMP1-mediated rosetting, while non-O groups—particularly type A—are associated with elevated risk and warrant enhanced clinical surveillance (Opi et al., 2023). In the Duffy system, the Fy(a-b-) phenotype confers near-complete resistance to Plasmodium vivax infection, though increasing reports of Duffy-independent invasion underscore the need for ongoing vigilance (Maheshwari et al., 2025). Within the MNSs system, glycoprotein receptors (GYPA/GYPB/GPC) facilitate merozoite invasion, yet naturally occurring variants such as Gerbich-negative and Dantu exert protective effects via biomechanical membrane alterations and reduced adhesion receptor density (Leffler et al., 2017; Jaskiewicz et al., 2018). Additional systems, including Knops (CR1) and CD36, modulate pathogenesis through immune-adhesive mechanisms and complement regulation (Leffler et al., 2017). Collectively, these findings support a blood group-informed risk stratification framework in endemic settings: high-risk phenotypes (e.g., non-O, Duffy-positive) should be prioritized for preventive resources and close monitoring, while individuals with protective genotypes (e.g., group O, Duffy-negative, Dantu/Ge (–)) may be strategically enrolled in studies of breakthrough infection or vaccine efficacy. Such a genetically nuanced approach may pave the way for precision public health strategies in malaria control.

Host genetic variation in blood group systems critically informs the development of malaria vaccines by revealing both viable targets and inherent challenges. The PfRh5–basigin (Ok blood group) interaction represents a leading vaccine candidate due to its essential role in erythrocyte invasion and limited polymorphism, facilitating broad neutralization across diverse Plasmodium falciparum strains (Alanine et al., 2019; Moore et al., 2021). In contrast, glycoprotein receptors such as GYPA (MNSs system) exhibit high allelic diversity and strain-specific binding, constraining the efficacy of vaccines targeting these pathways (McQuaid and Rowe, 2020). Beyond invasion, the ABO system inspires anti-disease strategies: natural protection in group O individuals through reduced PfEMP1-mediated rosetting supports immunogen design to mimic this phenotype and alleviate microvascular sequestration (Wassmer and Carlton, 2016). Furthermore, polymorphisms in CR1 (Knops blood group) influence both cytoadhesion and complement regulation, underscoring the need to account for host genetics in clinical trial design and immune response evaluation. Combining structural and population data on blood group antigens may help design better malaria vaccines that work across diverse human populations and evolving parasites.

In malaria-endemic regions, blood group polymorphisms significantly shape transfusion practices and safety outcomes. Protective variants—including group O in the ABO system, which mitigates severe malaria by impairing PfEMP1-mediated resetting (McQuaid and Rowe, 2020), and the Duffy-negative phenotype (Fy(a−b−)) (Duffy et al., 2023), which confers near-complete resistance to Plasmodium vivax invasion—provide a mechanistic rationale for selecting resistant blood components for specific clinical settings, such as exchange transfusion in cerebral malaria or transfusion support in P. vivax-endemic zones (Tan et al., 2013). However, implementing such targeted transfusion strategies faces considerable obstacles: a high frequency of asymptomatic parasitemia among donors sustains the risk of transfusion-transmitted malaria (TTM), while resource constraints often limit the implementation of sensitive screening methods in endemic areas (Keating et al., 2021; Harley and Tonetti, 2022). Moving forward, enhancing donor screening protocols, standardizing blood group and outcome documentation, and promoting interdisciplinary collaboration are essential to translating host genetic resistance into safe, evidence-based transfusion guidelines for malaria.

Yet major gaps remain. Most studies are confined to Africa and Southeast Asia, with scarce data from Latin America, Oceania or temperate imported cases. The spread of CRISPR/Cas9, organoids and high-throughput single-cell technologies now enables validation of candidate loci across the entire gene-phenotype-disease continuum, moving beyond simple epidemiological associations. In the coming years, integrating blood group genotyping into severe malaria early warning algorithms and embedding it within vaccine trials could advance individualized prevention. Concurrently, developing structure-guided PfEMP1-glycan or receptor blockers, and CRISPR/Cas9-engineered erythrocytes (Hawksworth et al., 2018; Boccacci et al., 2025), may accelerate malaria related clinical and basic research. As precision medicine converges with synthetic biology, blood groups will outgrow their role as clinical transfusion tags and become powerful tools in malaria prevention and treatment, bringing humanity one step closer to the ultimate goal of eradicating this ancient scourge.
